# The Paradox of Nuclear Lamins in Pathologies: Apparently Controversial Roles Explained by Tissue-Specific Mechanobiology

**DOI:** 10.3390/cells11142194

**Published:** 2022-07-13

**Authors:** Enrica Urciuoli, Barbara Peruzzi

**Affiliations:** Multifactorial Disease and Complex Phenotype Research Area, Bambino Gesù Children’s Hospital, IRCCS, 00165 Rome, Italy; enrica.urciuoli@opbg.net

**Keywords:** lamins, cancer, tissue mechanobiology, in vitro experimentation, stiffness

## Abstract

The nuclear lamina is a complex meshwork of intermediate filaments (lamins) that is located beneath the inner nuclear membrane and the surrounding nucleoplasm. The lamins exert both structural and functional roles in the nucleus and, by interacting with several nuclear proteins, are involved in a wide range of nuclear and cellular activities. Due their pivotal roles in basic cellular processes, lamin gene mutations, or modulations in lamin expression, are often associated with pathological conditions, ranging from rare genetic diseases, such as laminopathies, to cancer. Although a substantial amount of literature describes the effects that are mediated by the deregulation of nuclear lamins, some apparently controversial results have been reported, which may appear to conflict with each other. In this context, we herein provide our explanation of such “controversy”, which, in our opinion, derives from the tissue-specific expression of nuclear lamins and their close correlation with mechanotransduction processes, which could be very different, or even opposite, depending on the specific mechanical conditions that should not be compared (a tissue vs. another tissue, in vivo studies vs. cell cultures on glass/plastic supports, etc.). Moreover, we have stressed the relevance of considering and reproducing the “mechano-environment” in in vitro experimentation. Indeed, when primary cells that are collected from patients or donors are maintained in a culture, the mechanical signals deriving from canonical experimental procedures of cell culturing could alter the lamin expression, thereby profoundly modifying the assessed cell type, in some cases even too much, compared to the cell of origin.

## 1. Introduction

The nuclear lamina is a fibrillar meshwork of type-V intermediate filaments, termed lamins, that are important determinants of nuclear structure and functionality [1,2]. In eukaryotic cells, the nuclear lamins interact with the inner nuclear membrane proteins, nuclear pores, and other nucleoplasmic factors [3] and are directly connected to the peripheral chromatin in lamina-associated domains (LADs) [4].

Beside their expression levels, the nuclear lamin functions are determined by a plethora of post-translational modifications, including farnesylation, phosphorylation, acetylation, sumoylation, methylation, ubiquitination, and O-GlcNAcylation, that impact lamin activity, protein stability, and their interaction with associated proteins [5].

Historically, lamins have long been described solely as structural proteins that are involved in maintaining the nuclear shape and mechanics, but the scientific literature of the last two decades demonstrates lamins’ functional roles in regulating gene expression and chromatin organization, as well as DNA replication and repair and cellular signaling, as mechanotransduction processes [6,7].

The process of mechanotransduction implies that cells can perceive, integrate, and translate an external mechanical stimulation into a biochemical signal that elicits specific cellular responses, thereby allowing the cells to adapt to the continuous modifications that arise from the surrounding microenvironment [8]. The mechanical cues, depending on both the extracellular matrix stiffness/composition and the neighboring cells, are received by the cells through the mechano-sensitive structures, such as the focal adhesions, integrins, and the transmembrane receptor proteins [9], and are transduced intracellularly by mechano-responding factors, such as FAK [8,10], RhoA [11], Wnt [12], and YAP [13]. The activation of these pathways links the external ECM to the cytoskeleton, which, in turn, is connected to the nucleus by the LINC complex [14]. The intracellular cascade ends in the nucleus, where the lamins are primarily involved in orchestrating the ultimate translation of the external mechanical stimuli into the cellular response, mainly by modifying the nuclear structure [15,16], by modulating the gene expression through chromatin remodeling [6] and transcription factor regulation [17], and by reorganizing the cytoskeleton [18] according to the specific mechanical stimulation that is received [19,20].

Given the multifunctional roles that are exerted by nuclear lamins, any deregulation in their expression, gene sequence, and/or function leads to a wide spectrum of pathological conditions, ranging from genetic diseases to oncogenic transformation [21]. Laminopathies are rare genetic disorders with a varied phenotypic expression that is associated with more than 15 syndromes, which are categorized into five partially-overlapping phenotypic groups as follows: muscular dystrophies, neuropathies, cardiomyopathies, lipodystrophies, and progeroid syndromes [22]. More than 450 distinct mutations have been identified on the human *LMNA* gene, and the hallmark of laminopathies is the presence of nuclear abnormalities, including herniations, honeycomb-structures, and donut-like nuclei [23]. Apart from the similarities in an abnormal nuclear structure, a whole mechanistic characterization of *LMNA* gene mutations and the cell/tissue-specific functional effects is complex and still lacking in research. As an example, in a review article entitled “*The Broad Spectrum of LMNA Cardiac Diseases: From Molecular Mechanisms to Clinical Phenotype*”, Crasto and co-authors have declared that, despite the plethora of studies describing lamins’ involvement in many nuclear and cellular processes, none of these are capable, per se, in fully justifying the functional and clinical phenotypes of lamin-dependent cardiomyopathy [24], shedding light on the lack of knowledge in linking specific *LMNA* gene mutations to the distinct cell- or tissue-specific clinical manifestations.

A milestone in the functional characterization of lamins has been reached thanks to the work of Discher’s group, through an elegant demonstration that lamin-A expression scales with tissue stiffness, therefore being specifically related to the tissue mechano-environment [25]. Given the generalized relevance of this watershed document, the functions of the nuclear lamina have been reviewed by researchers working in any field of investigation that considers their role as mechano-sensors and their crucial involvement, not only in the nucleoskeleton structure, but also in the basic cellular processes. As a result, in recent years a plethora of scientific studies on the deregulated expression of the nuclear lamins in almost all pathological conditions have been published in the literature, also providing conflicting results, especially in the field of cancer research. Here, we provide our explanation of some of the controversies about lamins in cancer by considering the often-forgotten tissue-specificity of their expression. It is important to specify that, although this work is focused on lamin expression in relation to the tissue-specific mechano-environment, readers must keep in mind that the mechanotransduction process involves a plethora of proteins, other than lamins, and that lamin-dependent functions are not only determined by their gene expression, but also by post-transcriptional modifications, as well as the lamin-mediated activation of transcription factors and related proteins. In order to make this question less complex, we have exploited the lamin expression as a parameter to investigate how the cells perceive the extracellular mechano-environment, on the basis of Discher’s work. Furthermore, we have also underlined the relevance of considering and reproducing the tissue-specific mechano-environment in in vitro studies, without which the experimentation on cell culture may not be useful, due to the great divergence from in vivo conditions. 

## 2. Lamin Expression in Cancer: Tumor Suppressor or Oncogene?

The nuclear lamins control several critical cellular functions [26,27] and cancer cells frequently show a highly variable expression of lamin A/C, in comparison to the normal counterpart, therefore, the deregulation of lamins has been correlated to tumor onset and/or progression [28,29,30]. When conducting a review of the literature on the subject of “lamins and cancer”, these nuclear proteins, especially A-type, are described as oncogenes in some studies and as a tumor suppressors in other studies, making it difficult to obtain a comprehensive definition of their functional effects in cancer cells [29]. For example, the expression of lamin A/C correlates with increased cell mobility in a pre-metastatic colon adenocarcinoma cell line and is considered to be a poor-prognosis biomarker for colorectal cancer patients, given that its expression increases cell invasiveness and a more stem cell-like phenotype [31,32]. Pei et al. proposed nuclear lamins as potential oncogene targets for the therapeutic treatments of pediatric brain tumors. They asserted that all three of the human genes encoding for lamin isoforms (*LMNA*, *LMNB1*, and *LMNB2*) are significantly upregulated in glioma tissues compared with normal brain tissues and their silencing dramatically suppresses glioma progression in both in vitro and in vivo mouse models [33]. In parallel, Gatti and coworkers put forward lamin A/C as a biomarker of the aggressiveness and tumorigenicity in glioblastoma multiforme, as the expression of this nuclear protein was found to be correlated to a reduced overall survival rate in glioma patients and was mainly involved in cell adhesion and cell migration processes [34].

Kong and colleagues have described an increased expression of lamin A/C in prostate cancer tissues and have highlighted that in vitro overexpression of lamin A/C in DU145 and PC3 prostate cancer cells leads to enhanced migration and cell-invasion capabilities [35]. Recently, the same group have shown that the expression of lamin A/C varies within the same type of tumor, depending on the prostate cancer cell differentiation, as decreased levels of lamin A/C are associated with the epithelial-to-mesenchymal transition (EMT) process, while the re-expression of lamin A/C better correlates with the mesenchymal-to-epithelial transition (MET) process [36]. This study clearly demonstrated that lamins could serve as biosensors for cell differentiation status within the same type of cancer.

The aforementioned role that is exerted by lamin A/C as a cancer-favoring factor is completely overturned in neuroblastoma, which is the most common type of extracranial solid tumor that is observed in childhood. In fact, D’Agnano group’s work showed that a decreased level of lamin A/C in the high lamin A/C-expressing SH-SYS5 cell line blocks the retinoic acid-induced differentiation and allows the development of tumor-initiating cells, resulting in a more aggressive phenotype [37,38]. In view of this, lamin A/C behaves similar to an onco-suppressor factor in this type of cancer. In parallel, in breast cancer biopsies, higher levels of A-type lamins and *LMNB1* mRNA were detected in non-cancerous tissue in comparison to cancer tissue and, among such cancer tissues, the patients with a detectable lamin A/C expression were associated with better clinical outcomes than the patients with low lamin A/C expression [39]. Similarly, in ovarian cancer, the expression of lamin A/C is lower in cancer tissues in comparison to normal ovarian tissues and in benign tumor biopsies. In addition, the Kaplan–Meier analysis demonstrated that the patients with high lamin A had significantly longer overall survival and progression-free survival than the patients with low lamin A [40].

In the context of lamin A/C as onco-suppressor factor, our group has demonstrated that the expression of nuclear lamin A/C is inversely related to tumor aggressiveness in pediatric osteosarcoma cell lines [41], also demonstrating that the re-introduction of lamin A/C expression in the high-metastatic osteosarcoma cell line 143B reduces tumor aggressiveness and influences the adhesion properties on specific matrices [42]. Equally, Chiarini et al. has demonstrated a significant inverse correlation between *LMNA* gene expression and tumor aggressiveness and a poor prognosis in patients who are affected by Ewing sarcoma. These results were confirmed by *LMNA* overexpression in EWS cell lines, highlighting the role of lamin A as a tumor suppressor [43]. An overview of the aforementioned lamin expression in cancers is reported in Table 1.

In our opinion, based on Discher group’s work [25], these apparently conflicting results should not be surprising, since they were derived from studies on cancer cells originating from different tissues, in which the basal expression of lamins is very different, due to the mechanical properties of the microenvironment and the fact that each specific oncogenic transformation can alter their expression in different, and even opposite, ways.

## 3. Lamins and Their Role in the Tropism of Metastatic Cancer Cells: The Mechano-Environment Aspect in the “Seed and Soil” Theory

The classical “seed and soil” theory, which was formulated by Stephen Paget in 1889 and was revised by James Ewing in 1929, has provided the fundamental guidelines for research in cancer cell metastatization. In this theory, especially in the Ewing version, the involvement of mechanical issues is considered, proposing that metastatic dissemination occurs by purely mechanical factors that are determined by the anatomical structure of the vascular system, which dictates the site for metastatization. Nowadays, the seed and soil theory is still valid and accepted by the scientific community, although it has needed a revision with updated notions, in which the original term “seed” now comprises the afterwards identified progenitor, initiating, and cancer stem cells, and the term “soil” represents all of the aspects of the microenvironment in the metastasized organ. Regardless of the use of original or updated terminology, the relevance of the cross-talk between cancer cells and the receptive tissues in the outcome of the metastatic process is undoubted. In this context, we herein propose the consideration of the intrinsic expression of lamins in highly-aggressive cancer cells, which are usually different from normal cells, as a factor that is involved in the tropism of metastatization. More specifically, when an oncogenic transformation occurs in cells, the ratio between the lamin A/C and the lamin B can be altered, or even reversed, leading to a mechano-incompatibility of those cells in the surrounding microenvironment. Since lamins are involved in several key cellular processes, such as gene expression, migration, and cytoskeletal organization, it is reasonable to assume that their deregulation can contribute to an oncogenic behavior, triggering the metastatic process. Indeed, we have recently demonstrated that the aggressiveness of the cell lines deriving from pediatric osteosarcomas is inversely related to lamin A/C expression, and that the high-metastatic cell line 143B expresses low levels of lamin A/C, which is an unusual condition for cells of bone origin, in which the basal lamin A/C expression is typically very high [41]. Interestingly, these metastatic cells exhibit a ratio of lamin A/C:lamin B that is better suited to a softer tissue than bone, and indeed they metastasize to the lungs. Notably, parental 143B cells prefer to adhere on matrices of 1.5 kPa, resembling lung parenchyma, rather than 28 kPa matrices, resembling bone osteoid. This behavior is completely reversed when lamin A/C expression is restored by plasmid transfection [42]. Another example in support of our hypothesis is represented by the lamin expression in breast cancer cell lines. Low-aggressive MCF-7 cells express low levels of lamin A/C, a condition that fits with the stiffness of the breast tissue, and indeed they stay in the breast and do not metastasize. On the contrary, high-metastatic MDA-MB-231 cells show an increased expression of lamin A/C, a condition that is more suited to bone tissue, and indeed, they preferentially metastasize to the bone [44,45]. Remaining in the context of breast cancer, it would be very interesting to assess the lamin A/C expression in high-metastatic cancer cells with a tropism toward a softer tissue than bone, for example, the MDA231-Brm2 cell line that metastasize preferentially to the brain (to the best of our knowledge, there are no papers in literature showing lamin expression in these cells). According to our hypothesis, these cells, although high-metastatic, as well as bone-tropic MDA-MB-231 cells, should express very low levels of lamin A/C, that should make them suitable for the brain mechano-environment, which is a softer tissue in comparison to breast.

At the same time, given that lamins are key pivotal mechano-sensors, a deregulation in lamin expression may be related to a loss of mechano-sensitivity, a crucial signal for the maintenance of tissue homeostasis. Once again, the lack of perceiving and transducing external mechanical signals could induce cell departure from a hostile environment. In support of this, the new term “mechanobiome” was coined in order to shed light on the relevance in considering all of the aspects of tissue mechanobiology, the mechanotransduction process, and the mechano-environment when studying cancers [46].

## 4. Discrepancy in Lamin Expression between In Vivo and In Vitro Experimentations

Several works have recently explained the role of nuclear lamins in providing a mechanical support to the nucleoskeleton, thereby recognizing these proteins as regulators of gene expression and the mechanical sensors for tissue elasticity. Numerous studies have demonstrated that lamin-A expression functions as a “mechanostat”, revealing that matrix stiffness and mechanical stress are able to regulate lamin-A expression, thereby stabilizing the nucleus and contributing to cell lineage determination [25,47,48,49]. Once again, the work by Discher’s group has clearly demonstrated that mechanical issues should be considered where in vitro experimentation is conducted. Indeed, human mesenchymal stromal cells can differentiate into osteogenic lineage when they are cultured on a stiff matrix, without the need of the canonical treatment with a differentiation medium, which is commonly used by the scientific community [25]. On the contrary, the same mesenchymal stromal cells differentiate into adipogenic lineage if they are cultured on a soft matrix, and this stiffness-dependent lineage specification is driven by lamin A/C expression [36]. In our opinion, this study has demonstrated that in vitro experimentation needs to consider and recapitulate the mechano-environment of the origin tissue by which the cultured cells are collected.

As discussed above, the alterations of nuclear lamin expression are strictly related to the different types of cancer and their aggressiveness and this variability is due to the specific stiffness of the tissue of the cancer origin and the relative tumor microenvironment.

The complexity of studying lamin expression increases by comparing in vitro and in vivo studies related to the same tissue, given that some works have revealed controversial results about lamin expression that were assessed in primary cancer tissue in comparison to cultured cancer cell lines. For instance, Alhudir et al. evaluated lamin A/C expression by immunohistochemistry in a tissue microarray containing 938 early-stage breast cancer biopsies, finding that reduced lamin A/C correlates with the development of distant metastases, a poor prognosis, and a shorter overall survival [50]. Indeed, as described above, when lamin A/C expression is investigated in breast cancer commercial cell lines that are cultured in canonical plastic (polystyrene) dishes, we find a higher expression of high-metastatic MDA-MB-231 cells in comparison to low-aggressive MCF-7 cells [44,45], which is the opposite of that which is described in primary tissues. Moreover, it has been found that the in vitro suspension state was able to increase lamin A/C accumulation in MDA-MB-231 cells and the following lamin A/C knockdown also decreased drug resistance [51] and cell migration [52]. Another example refers to the lamin A/C expression in macrophages, which is different if it is assessed in the primary tissue resident macrophages in comparison to the immortalized macrophage cell line (RAW-264.7). Indeed, as declared by Mehl and coworkers, this contradictory observation might not be surprising, as multiple papers highlight significant cell signaling differences between the primary and the RAW-264.7 macrophages [53,54].

Taking into account that any in vitro experimental condition cannot fully reproduce the in vivo environment, and that the best way to perform experiments is to use appropriate controls and multiple approaches to the same biological issue, in our opinion, most of the aforementioned discrepancies can be explained by considering the experimental procedures. In fact, commercial cell lines suffer abnormal mechanical stimuli when they are cultured into the polystyrene dishes with a stiffness in the order of GigaPa, while human tissue matrices are in the order of kPa. This in vitro mechanical stimulation mediated by a stiff polystyrene substrate can alter the lamin expression in cultured cells, thereby inducing modulation in gene transcription, as well as in a plethora of other cellular functions.

## 5. Conclusions

In this opinion article, we have provided our point of view about the importance and the need of considering mechanical issues in scientific experimentation and result interpretation, especially in the context of lamin mechano-sensors and cancer research.

Although lamin expression and activity are regulated by several upstream signals that are dependent on the mechanotransduction process, and are mainly related to the cell differentiation status [55,56], starting from the work by Swift and co-authors, nuclear lamins became a generalized mechano-sensor that is needed in order to monitor the changes in the mechanobiology of a specific tissue or group of cells that are isolated from a specific tissue. Indeed, when nuclear lamins are investigated, researchers must keep in mind that each human tissue exhibits a specific lamin ratio and, therefore, should not be misled by some seemingly conflicting results, as shown by the systematic review of the literature. Moreover, we have also stressed the relevance of recapitulating the in vivo mechano-environment in in vitro experimentation, by providing clear examples of discordant results on lamin expression in comparison between the primary tissue and the in vitro cultured cell lines.

## Figures and Tables

**Table 1:** Lamin expression in primary cancer tissues and in cancer cell lines. Referring to the text, for each cancer type we have specified the samples assessed in the relative work, the basal lamin expression, and how in vitro modification of lamin expression impacts the tumoral behavior of cancer cell lines. ↑ = increase. ↓ = decrease.

Cancer Type	Samples	Basal Lamin Expression	In Vitro Modification (Cancer Cell Lines)	Effects on Cells Mediated by Lamin in Vitro Modification	Ref
**Colon adenocarcinoma**	Normal colonic Mucosa	Lamin A expressed in the basal region of colonic crypts (stem cell niche)			[32]
	Cancer tissue	Worse prognosis for A-type lamin-expressing tumors			
	SW480 cell line	Lamin A/C Undetectable	Over-expression of Lamin A by stable transfection	↑ cell motility and stem cell-like phenotype	
**Glioma**	Primary surgical Specimens	*LMNA, LMB1,* and *LMNB2* genes are upregulated in glioma tissue compared with normal brain tissue			[33]
	U87-MG and U251 glioma cell lines	A- and B-type lamins detected	Silencing of *LMNA, LMNB1,* and *LMNB2* genes by shRNA in cell line	↓ growth and cell mobility of glioma cells. Regular nuclear shape restored	
**Glioblastoma Multiforme**	Primary GBM Tumors	Expression of *LMNA* gene correlates with reduced overall survival			[34]
	T98G GBM cell line	Moderate expression of lamin A/C, comparable to normal astrocytes	Over-expression of *LMNA* gene	↑ cell aggressiveness and migratory ability	
			Silencing of *LMNA* gene	↓ cell aggressiveness and migratory ability	
**Prostate cancer**	Benign and malignant prostate tissue	High lamin A/C in benign glands; weak lamin A/C in low-grade tumors; high lamin A/C in high-grade tumors			[35]
	PC3, DU145, and LNCaP PC cell lines	Lamin A/C detected	Over-expression of *LMNA* gene	↑ cell growth and colony formation	
			Silencing of *LMNA* gene	↓ cell growth and colony formation	
**Neuroblastoma**	Biopsies	*LMNA* gene expression inversely related to MYCN expression			
	SH-SY5Y cell line	High Lamin A/C expression	*LMNA* knock-down	↑ cell motility and invasion, tumor initiating cells developed, differentiation blocked	
**Breast** **cancer**	Normal breast and cancer tissues from patients	High levels of A-type lamin in non-cancerous tissue. Higher A-type lamin expression associated with better clinical outcome			[39]
**Ovarian cancer**	Ovarian cancer tissues	High expression of lamin-A associated with better survival			[40]
	High-metastatic HO-8910 cell line	Lamin A/C Detected	Over-expression of *LMNA* gene	↓ cell migration	
			Silencing of *LMNA* gene	↑ cell migration	
**Osteosarcoma**	Specimens from osteosarcoma patients	Lamin A/C expression positively correlated with overall survival			[41,42]
	Normal human osteoblasts, SaOS-2, HOS, and 143B cell lines	Lamin A/C expression inversely related to aggressiveness	Silencing of *LMNA* gene in low-aggressive SaOS2 cell line	↑ cell proliferation	
			Over-expression of *LMNA* gene in metastatic 143B cell line	↓ tumor aggressiveness ↑ adhesion on stiffer matrix	
**Ewing Sarcoma**	Primary tumors and metastasis	Inverse correlation between *LMNA* gene expression and tumor Aggressiveness			[43]
	TC-61 and A-673 cell lines	Higher Lamin A/C expression in A-673 cell line in comparison to TC-61 cells.	Silencing of *LMNA* gene in A-673 cell line	↑ cell aggressiveness	
			Over-expression of *LMNA* gene in TC-71cell line	↓ motility, migration, and metastatic load in the liver	

## Data Availability

Not applicable.
